# Preparation and Performance Study of Boron Adsorbent from Plasma-Grafted Polypropylene Melt-Blown Fibers

**DOI:** 10.3390/polym16111460

**Published:** 2024-05-22

**Authors:** Yi Qin, Hui Jiang, Zhengwei Luo, Wenhua Geng, Jianliang Zhu

**Affiliations:** 1 College of Biotechnology and Pharmaceutical Engineering, Nanjing Tech University, Nanjing 211816, China; 202161218099@njtech.edu.cn (Y.Q.); huijiang@njtech.edu.cn (H.J.); jlzhu@njtech.edu.cn (J.Z.); 2 School of Environmental Science and Engineering, Nanjing Tech University, Nanjing 211816, China; luozw2015@njtech.edu.cn

**Keywords:** plasma graft polymerization, PP melt-blown fibers, boron, adsorption

## Abstract

In this study, the plasma graft polymerization technique was used to graft glycidyl methacrylate (GMA) onto polypropylene (PP) melt-blown fibers, which were subsequently aminated with N-methyl-D-glucamine (NMDG) by a ring-opening reaction, resulting in the formation of a boron adsorbent denoted as PP-g-GMA-NMDG. The optimal conditions for GMA concentration, grafting time, grafting temperature, and the quantity of NMDG were determined using both single factor testing and orthogonal testing. These experiments determined the optimal process conditions to achieve a high boron adsorption capacity of PP-g-GMA-NMDG. Fourier-transform infrared spectroscopy (FT-IR), scanning electron microscopy (SEM), energy dispersion spectrum analysis (EDS), and water contact angle measurements were performed to characterize the prepared adsorbent. Boron adsorption experiments were carried out to investigate the effects of pH, time, temperature, and boron concentration on the boron adsorption capacity of PP-g-GMA-NMDG. The adsorption isotherms and kinetics of PP-g-GMA-NMDG for boron were also studied. The results demonstrated that the adsorption process followed a pseudo-second-order kinetic model and a Langmuir isothermal model. At a pH of 6, the maximum saturation adsorption capacity of PP-g-GMA-NMDG for boron was 18.03 ± 1 mg/g. In addition, PP-g-GMA-NMDG also showed excellent selectivity for the adsorption of boron in the presence of other cations, such as Na^+^, Mg^2+^, and Ca^2+^, PP-g-GMA-NMDG, and exhibited excellent selectivity towards boron adsorption. These results indicated that the technique of preparing PP-g-GMA-NMDG is both viable and environmentally benign. The PP-g-GMA-NMDG that was made has better qualities than other similar adsorbents. It has a high adsorption capacity, great selectivity, reliable repeatability, and easy recovery. These advantages indicated that the adsorbents have significant potential for widespread application in the separation of boron in water.

## 1. Introduction

Boron is widespread in the Earth’s rock strata and is one of the most important elements in the Earth’s crust. It is not only found in boron minerals but also in natural waters, such as the brine of salt lakes, seawater, and geothermal water [1,2]. Boron is commonly found in nature as compounds, such as borate and borosilicate [3]. Boron compounds exhibit multiple oxidation states, with the +3 valence state being the most common. Hypervalent borates are predominantly observed in low oxidation states, such as +1, 0, or negative valence states.

Boron and its compounds usually have special properties such as lightness, flame retardancy, heat resistance, and abrasion resistance, making them highly sought after in various industries such as electronics, medicine, and agriculture [4,5,6,7]. At the same time, boron, as a trace element, plays many useful roles in the biological metabolism and physiological processes of plants and animals [8]. In most plants, boron promotes the growth and expansion of plant roots and increases the sugar content of fruit [9]. In humans, boron promotes the growth of bones, blood vessels, and the brain [10,11]. However, the production, application, and development of boron resources in the boron industry generate boron-containing waste, which leads to environmental pollution and waste of boron resources. When boron accumulates in soil and groundwater, excess boron in irrigation or drinking water can cause cell damage or toxicity to both humans and various species of flora and fauna [12]. The World Health Organization recommends a permissible concentration limit of 2.4 mg/L for drinking water, while China has set its standard at 1.0 mg/L [13]. As conventional methods of removing boron from drinking water sources fail due to the surrounding geology and wastewater discharge, it is of great practical importance to find an efficient yet cost-effective method that removes or recovers this resource from the aquatic environment.

Currently, the removal and recovery methods of boron include chemical precipitation, extraction, membrane separation, ion exchange, electrophoresis, and adsorption [14,15,16,17,18]. Among these methods, adsorption is considered an ideal approach for deep boron removal due to its simplicity, low cost, excellent boron adsorption performance, and ease of regeneration [19]. To produce the effect of boron absorption, the adsorption approach uses the cis-ortho-dihydroxyl of the functional group to form a combination with boric acid or borate ion. Functional groups include o-binary phenols [20], hydroxyl carboxylic acids, and N-methyl-D-glucosamine (NMDG) [21], of which NMDG is one of the most commonly employed. The NMDG molecule structure contains a secondary amine nitrogen and eight adjacent hydroxyl groups, and introducing NMDG into the polymer chain structure allows the multiple hydroxyl groups to chelate with boron, thereby achieving the selective adsorption of boron [22]. Commonly, metal skeletons [23], silicone resin [24], natural products [25], and other manufactured materials are used as adsorbents. Polypropylene (PP) melt-blown fibers are low-cost textile fibers with a small single-fiber diameter, high porosity, large surface area, outstanding chemical and microbial resistance, and ease of processing, making them widely used as matrix materials [26,27,28]. However, PP is a hydrophobic polymer, and its molecular chain lacks polar functional groups, which limits the application of boron removal and recovery in aqueous environments [29,30]. Therefore, surface modification of PP by introducing functional groups with a specific boron adsorption affinity is required.

Graft polymerization has been recognized as one of the most effective methods for polymer surface modification, which can be achieved through chemical initiators, ultraviolet light, plasma, or ionizing radiation [31,32]. Due to the merits of efficiency, cleanliness, environmental friendliness, and the non-destructive nature of material internal structures, plasma has been widely applied in chemical and physical surface modifications [33,34,35]. However, the surface modification of polymers, for example, wettability, endowed with solely plasma treatment, will disappear since the active sites are not stable, which is known as the aging effect or hydrophobic recovery [36]. The grafting polymerization reaction triggered by plasma treatment can avoid the limitation and help the modified materials maintain long-term stability. Cheng et al. [37] formed a hydrophilic acrylic acid coating on polytetrafluoroethylene (PTFE) surfaces using atmospheric pressure plasma-induced graft polymerization and investigated the effect of atmospheric pressure plasma treatment on the surface properties of PTFE. Haji et al. [38] used oxygen plasma to modify the hydrophilicity of PP nonwoven fabric followed by grafting *β*-cyclodextrin.

The purpose of this study is to convert polypropylene melt-blown fibers, a cheap and abundant polymer substrate, into efficient boron adsorbents using a simple and environmentally friendly procedure. Plasma treatment will be used to induce glycidyl methacrylate (GMA) graft polymerization and introduce NMDG onto the epoxy groups of the graft polymer chains, successfully modifying PP melt-blown fibers into boron adsorption fibers. Single-factor experiments and orthogonal experiments will be used to optimize the preparation process of GMA-grafted PP fibers. The optimal process conditions for preparing PP-g-GMA-NMDG with the highest boron adsorption capacity will be studied. The resultant fibers will be characterized, and their adsorption behavior under different conditions, adsorption mechanism, and selective adsorption ability will be investigated.

## 2. Experimental

### 2.1. Materials and Apparatus

PP melt-blown fibers were obtained from Jiangnan University. Methanol was obtained from Yonghua Chemical Co., Ltd. (AR, Suzhou, China). GMA was obtained from Shanghai Aladdin Biochemical Technology Co., Ltd. (AR, Shanghai, China), 1,4-dioxane was obtained from Shanghai Maclin Biochemical Technology Co., Ltd. (AR, Shanghai, China), and NMDG from Shanghai Yuanye Biotechnology Co., Ltd. (AR, Shanghai, China). The plasma generator used in this study was the VP-R series vacuum plasma processor (13.56 MHz) manufactured by Guangzhou SunJune Technology Co., Ltd. (Guangzhou, China).

### 2.2. Preparation of Adsorbent

#### 2.2.1. Pretreatment of PP Fibers

The PP melt-blown fibers were cut into square pieces and successively cleaned with acetone and deionized water. They were then dried in a vacuum drying oven at 60 °C to a constant weight and stored in a sealed desiccator for later use.

#### 2.2.2. Preparation of PP-g-GMA

The pretreated PP fibers (0.1 g) were placed in the plasma reaction chamber with the plasma power adjusted to 100 W and the gas flow rate set to 250 mL/min. Both sides of PP fibers were treated under different atmospheres (air, oxygen, and argon) and different durations (30~150 s). After plasma irradiation, PP fibers were quickly immersed in a degassed GMA-methanol solution of certain concentrations (10–80%) and containing 0.5 g of Mohr’s salt (anti-agglomerant). Graft polymerization was carried out in a constant-temperature water bath at a certain temperature (55–75 °C) for a specific time (30–180 min). The resulting product was sequentially washed with acetone and deionized water, then dried in a vacuum drying oven at 60 °C to a constant weight.

#### 2.2.3. Preparation of PP-g-GMA-NMDG

**Figure 1:**
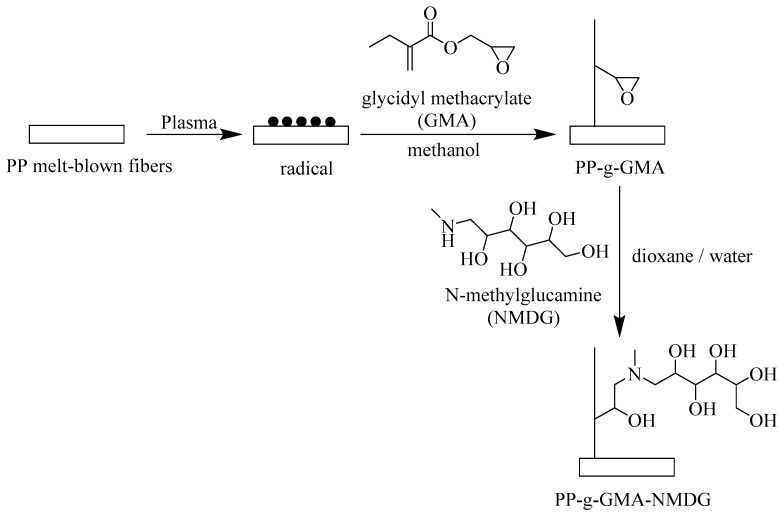
Synthesis roadmap of PP-g-GMA-NMDG.

First, a certain amount of NMDG (2–4.5 g) was dissolved in 10 mL of deionized water, followed by the addition of 40 mL of 1,4-dioxane. A certain amount of PP-g-GMA fiber was immersed in the solution and then mechanically stirred at 70 °C for 10 h. After the reaction, the obtained PP-g-GMA-NMDG fibers were washed with deionized water, then dried in a vacuum-drying oven at 60 °C to a constant weight. The preparation process of PP-g-GMA-NMDG is illustrated in Figure 1.

### 2.3. Characterizations

The structure of the materials was analyzed using a Fourier-transform infrared spectrometer (FT-IR, Nicolet 6700, Thermo Fisher, Waltham, MA, USA) within the range of 500–4000 cm^−1^. Scanning electron microscopy (SEM, Thermo Fisher, USA) was utilized to examine the surface morphology of PP, PP-g-GMA, and PP-g-GMA-NMDG. The hydrophilicity of fibers was analyzed using contact angle testing (Theta Flex, Stockholm, Sweden).

### 2.4. Adsorption Experiment of PP-g-GMA-NMDG

The adsorption performance of PP-g-GMA-NMDG was assessed in terms of pH, temperature, time, solution concentration, and adsorbent selectivity. The simulated boron solution was prepared, and the pH was adjusted using dilute NaOH or HCl. PP-g-GMA-NMDG fibers (0.02 g), which were then added to the boron acid solution, and adsorption was carried out with shaking at 200 rpm. Then, solution samples were taken at specific intervals and filtered using the filter heads (0.45 μm). The concentrations of boron before and after the adsorption were determined using an inductively coupled plasma-optical emission spectrometer (ICP-OES, iCAP 6300, Thermo Fisher, USA). The following formulas were used to calculate the boron adsorption capacity (*Q*_t_) and removal efficiency (*R*) of PP-g-GMA-NMDG fibers:

(1) $$Q_{t}=\frac{(C_{0}-C_{t})V}{m}$$

(2) $$R=\frac{C_{0}-C_{t}}{C_{0}}\times 100\%$$

where *Q_t_* is the boron adsorption capacity of PP-g-GMA-NMDG fibers at time *t*, mg/g; *C*_0_ is the initial mass concentration of boron, mg/L; *C_t_* is the mass concentration of boron at time *t*, mg/L; *V* is the volume of solution, L; and *m* is the mass of PP-g-GMA-NMDG fibers, g.

#### 2.4.1. pH

Boron solutions (200 mg/L) were prepared and adjusted to pH values of 2, 4, 6, 8, and 10. PP-g-GMA-NMDG fibers (0.02 g) were added to each solution in stoppered bottles and shaken (200 rpm, 25 °C) for 2 h. The concentrations of boron before and after the adsorption were determined, and the boron adsorption capacity was calculated.

#### 2.4.2. Adsorption Kinetics

Ten portions of boron acid solution with a concentration of 50 mg/L and a pH of 6 were prepared in stoppered bottles. PP-g-GMA-NMDG fibers (0.02 g) were added to each bottle, and adsorption was carried out at room temperature with shaking at 200 rpm for various time intervals (5, 10, 20, 30, 50, 70, 90, 120, 150, and 180 min). The concentrations of boron before and after the adsorption were determined, and the boron adsorption capacity was calculated. The pseudo-first-order and pseudo-second-order adsorption kinetics models were used to simulate the adsorption kinetics behavior of PP-g-GMA-NMDG.

(3) $$\log\left(Q_{e}-Q_{t}\right)=logQ_{e}-k_{1}t$$

(4) $$\frac{t}{Q_{t}}=\frac{t}{Q_{e}}+\frac{1}{k_{2}Q_{e}^{2}}$$

where *k*_1_ represents the rate constant of pseudo-first-order kinetics, min^−1^; *k*_2_ represents the rate constant of pseudo-second-order kinetics, g/(mg·min); *t* is the reaction time, min; and *Q_e_* and *Q_t_* represent the adsorption capacity at equilibrium and at time *t*, mg/g.

#### 2.4.3. Adsorption Isotherms

PP-g-GMA-NMDG (0.02 g) was added into boron solution (10 mL, pH = 6) with concentrations ranging from 5–500 mg/L, and the mixtures were shaken for 2 h (200 rpm, 25 °C). The concentrations of boron before and after the adsorption were determined, and the boron adsorption capacity was calculated. The adsorption isotherm of the PP-g-GMA-NMDG adsorbent was fitted using both Langmuir and Freundlich isotherm models. The Langmuir isotherm model assumes monolayer adsorption without energy variation and is applicable to various concentration conditions, primarily explaining the chemical adsorption process of monolayers. The Freundlich isotherm model, an empirical model, is mainly used to explain the multi-layer adsorption process [39].

The Langmuir adsorption isotherm equation is:

(5) $$Q_{e}=\frac{K_{L}Q_{m}C_{e}}{1+K_{L}C_{e}}$$

The Langmuir adsorption isotherm equation is:

(6) $$Q_{e}=K_{F}C_{e}^{1/n}$$

where *Q_e_* is the adsorption capacity of PP-g-GMA-NMDG for boron at equilibrium, mg/g; *K_L_* is the Langmuir adsorption equilibrium constant, L/mg; *K_F_* is the Freundlich adsorption equilibrium constant, mg/(g·mg^n^·L^n^); *C*_e_ is the boron mass concentration of the solution at equilibrium, mg/L; *Q_m_* is the saturation adsorption capacity of PP-g-GMA-NMDG for boron, mg/g; 1/*n* is the Freundlich index. *n* expresses the adsorption capacity parameter of the system, reflecting the adsorption performance of the adsorbent. Previous studies have shown that when 0.1 < 1/*n* < 0.5, the adsorbent can easily adsorb the target substance, while when 1/*n* > 2, the adsorbent struggles to adsorb the target substance [39].

#### 2.4.4. Adsorption Thermodynamics

PP-g-GMA-NMDG (0.02 g) was added into boron solution (10 mL, pH = 6) with concentration ranges from 50 to 500 mg/L, and the mixtures were shaken (200 rpm) for 2 h at temperatures of 25, 35, and 45 °C, respectively. The concentrations of boron before and after the adsorption were determined, and the boron adsorption capacity was calculated.

#### 2.4.5. Adsorption Selectivity

NaCl, MgCl_2_, and CaCl_2_ were added to a boron acid solution of 200 mg/L to prepare boron solutions with coexisting interfering ions of Na^+^, Mg^2+^, and Ca^2+^. Then, 10 mL of each prepared solution was taken in separate flasks, and 0.02 g of PP-g-GMA-NMDG was added to each flask. The mixtures were shaken at 25 °C (200 rpm) for 2 h. The concentrations of boron before and after the adsorption were determined, and the boron adsorption capacity was calculated.

#### 2.4.6. Reusability

PP-g-GMA-NMDG (0.02 g) was introduced into a boron solution (10 mL, pH = 6) with a concentration of 200 mg/L. The resulting mixture was agitated at 200 rpm for a duration of 2 h. The concentrations of boron were measured both before and after the process of adsorption, and the adsorption capacity of boron was then computed.

The regeneration method involved adding the adsorbent to a 1 mol/L HCl solution for elution. The mixture was then treated to ultrasonication for 30 min, followed by centrifugation to remove the clear liquid. Finally, the remaining material was neutralized using a 1 mol/L NaOH solution. The substance was then rinsed with distilled water until it reached a neutral pH, subjected to centrifugation once more to eliminate the transparent liquid, and finally dried in a vacuum drying oven. Subsequently, the adsorption experiment was carried out using same experimental conditions and procedures as previously stated.

## 3. Results and Discussion

### 3.1. Optimization of Graft Polymerization Conditions

#### 3.1.1. Plasma Treatment Atmosphere

**Figure 2:**
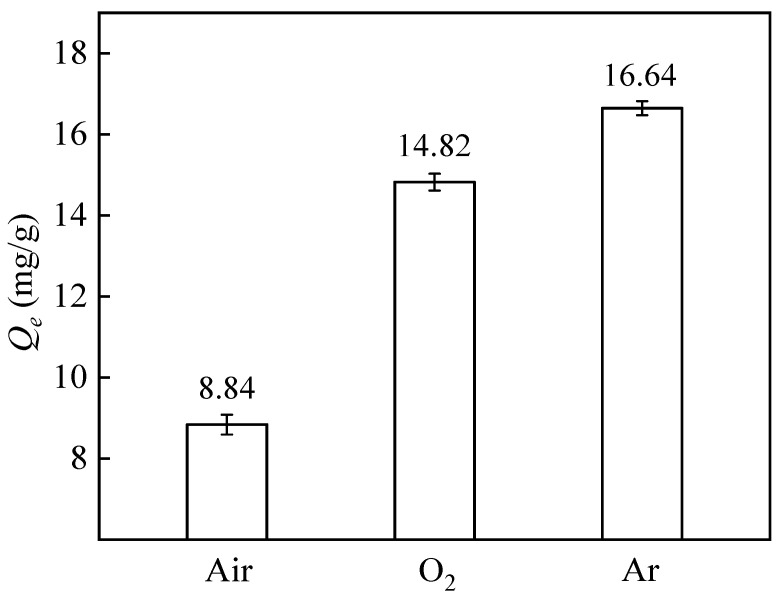
Influence of plasma treatment atmosphere on the adsorption capacity of PP-g-GMA-NMDG (plasma treatment time 90 s, 20% GMA, grafting temperature 70 °C, grafting time 150 min).

The influence of the plasma treatment atmosphere on the adsorption capacity of PP-g-GMA-NMDG is shown in Figure 2. The highest adsorption capacity of PP-g-GMA-NMDG was achieved with argon plasma treatment. This is because the argon plasma activates the free radicals on the surface of the PP fibers and increases the density of free radicals on their surface [39]. The presence of these free radicals promotes the attachment of additional GMA, resulting in the PP-g-GMA-NMDG fibers having an increased capacity to adsorb boron, with a maximum adsorption capacity of 16.64 mg/g. The lower pressure and concentration of the air lead to a lower probability of collisions between electrons and gas molecules, so that the plasma density is not sufficient. The higher reactivity of the oxygen atoms in pure O_2_ tends to react with the electrons and free radicals generated by the glow so that the plasma density in the reaction chamber is reduced [40]. Therefore, argon is chosen for the plasma treatment of PP fibers and has the best treatment effect.

#### 3.1.2. Plasma Treatment Time Subsubsection

**Figure 3:**
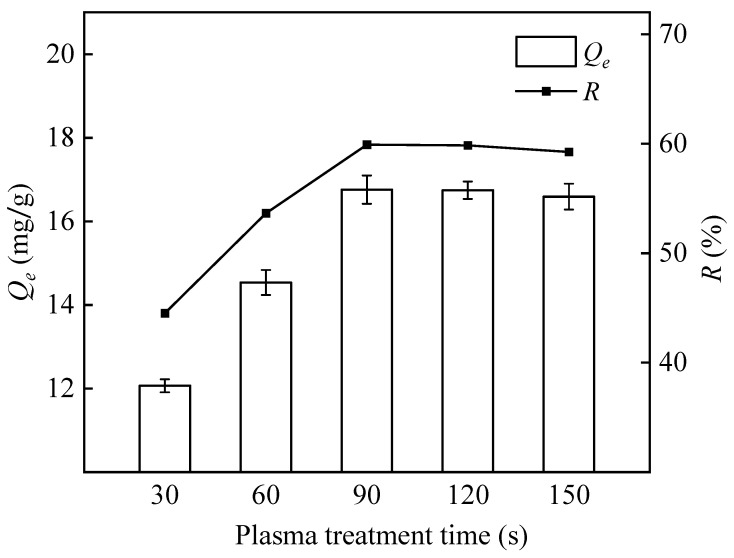
Influence of plasma treatment time on the adsorption capacity and removal rate of PP-g-GMA-NMDG (Ar plasma treatment, 20% GMA, grafting temperature 70 °C, grafting time 150 min).

Figure 3 shows that the adsorption capacity of PP-g-GMA-NMDG increases with the increasing duration of treatment with argon plasma and tends to stabilize after 90 s. After plasma treatment, reactive free radicals were formed on the surface of the PP fibers, possibly increasing the effectiveness of the subsequent graft polymerization [33]. The amount of free radicals generated on the surface of the PP fibers reaches a critical threshold when the treatment time reaches a certain value. Further extension of the treatment time at this stage increases the potential for radical complex formation and termination of the reaction, resulting in a stable number of incipient free radicals and slower polymerization [41]. Therefore, 90 s is the ideal time period for plasma treatment.

#### 3.1.3. Concentration of GMA

**Figure 4:**
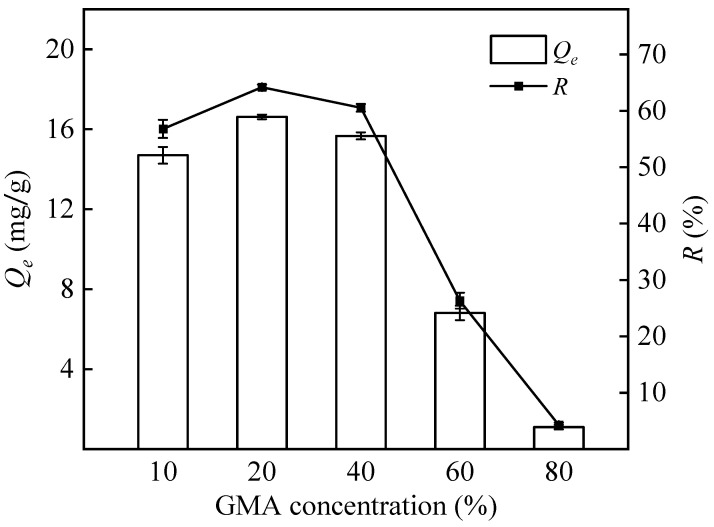
Effect of GMA concentration on the adsorption capacity and removal rate of PP-g-GMA-NMDG (Ar plasma treatment time 90 s, grafting temperature 70 °C, grafting time 150 min).

Figure 4 shows how the adsorption capacity of PP-g-GMA-NMDG increases and reaches a maximum of 16.61 mg/g when the concentration of GMA is between 10% and 20%. The adsorption capacity decreases when the GMA concentration increases above 20%. The reason is that higher monomer concentrations increase the collision probability of monomers and free radicals, which can activate graft polymerization [42]. Therefore, more monomer is grafted onto the surface of the PP fibers, increasing the amount of boron adsorbed by PP-g-GMA-NMDG. However, excessive monomer concentration increases the probability of rapid homopolymerization, raises the viscosity of the reaction system, and enhances the formation of reaction by-products, consequently diminishing the adsorption capacity and decreasing grafting efficiency [43]. In summary, a GMA concentration of 10–20% promotes the best graft polymerization.

#### 3.1.4. Graft Polymerization Time

**Figure 5:**
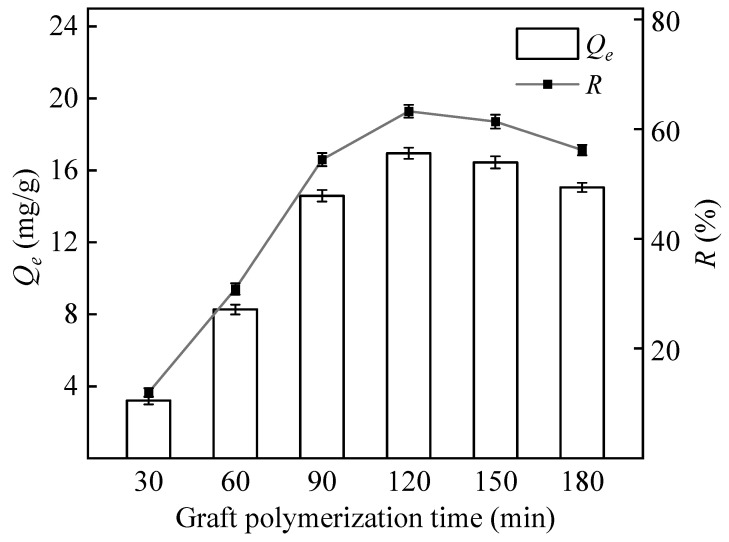
Effect of graft polymerization reaction time on the adsorption capacity and removal rate of PP-g-GMA-NMDG (Ar plasma treatment 90 s, 20% GMA, graft temperature 70 °C).

In the initial stages of the reaction, active free radicals on the surface of PP fibers react rapidly with monomeric GMA, leading to a rapid increase in boron adsorption capacity. As the graft copolymerization progresses gradually, the grafting effect subsequently decreases, as shown in Figure 5. Therefore, the optimal range for the reaction time is between 90 and 150 min.

#### 3.1.5. Graft Polymerization Temperature

**Figure 6:**
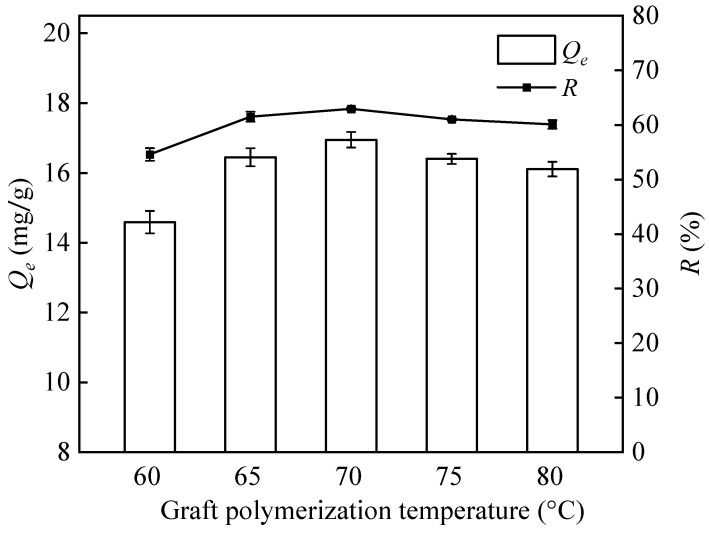
Effect of graft polymerization temperature on the adsorption capacity and removal rate of PP-g-GMA-NMDG (Ar plasma treatment time 90 s, 20% GMA, graft time 150 min).

As the temperature of the grafting reaction increases, the adsorption capacity and removal rate gradually increase (Figure 6). However, when the reaction temperature exceeds 70 °C, the adsorption capacity decreases. This is because the diffusion rate of GMA and the reactivity of the free radicals increase with temperature, resulting in an enhanced grafting effect at higher temperatures. However, excessively high temperatures accelerate the rate of decomposition of the free radicals and exceed the rate of grafting reactions. This intensifies the termination reactions of the free radicals and, at the same time, increases the synthesis of GMA homopolymers. This leads to an increased viscosity of the reaction mixture and a reduced flowability, which hinders the transfer and diffusion of the chains and thus impedes the grafting reaction [44]. Therefore, the optimum temperature range for graft copolymerization is 65 to 75 °C.

### 3.2. Orthogonal Experiment for Optimizing Combination Conditions

The influence of the plasma treatment atmosphere on the adsorption capacity of PP-g-GMA-NMDG is shown in Figure 2. The highest adsorption capacity of PP-g-GMA-NMDG was achieved with argon plasma treatment. This is because the argon plasma activates the free radicals on the surface of the PP fibers and increases the density of free radicals on their surface [39]. The presence of these free radicals promotes the attachment of additional GMA, resulting in the PP-g-GMA-NMDG fibers having an increased capacity to adsorb boron, with a maximum adsorption capacity of 16.64 mg/g. The lower pressure and concentration of the air lead to a lower probability of collisions between electrons and gas molecules, so that the plasma density is not sufficient. The higher reactivity of the oxygen atoms in pure O_2_ tends to react with the electrons and free radicals generated by the glow, so that the plasma density in the reaction chamber is reduced [40]. Therefore, argon is chosen for the plasma treatment of PP fibers, which has the best treatment effect.

#### 3.2.1. Determination of Experimental Factor Levels Table

In this study, GMA concentration, graft polymerization reaction time, and graft polymerization reaction temperature were selected as influencing factors, and the adsorption capacity of boron by PP-g-GMA-NMDG fibers served as an experimental index. An orthogonal experimental method with three factors at three levels was applied, and the orthogonal table L_9_(3^4^) was used to optimize the process parameters for the preparation of PP-g-GMA-NMDG fibers, as shown in Table 1 for the selection of factor levels.

#### 3.2.2. Analysis of Orthogonal Experimental Results

From the results in Table 2, it can be concluded that the PP-g-GMA-NMDG fibers prepared under A3B3C2 conditions exhibited the best adsorption efficiency for boron, with the highest adsorption capacity being 18.03 mg/g. Therefore, a GMA concentration of 20% and a reaction time of 150 min at 70 °C were set as the optimum experimental conditions for graft copolymerization. Analysis of the values shows that the factors affecting the preparation of PP-g-GMA-NMDG fibers are in the order of GMA concentration > graft polymerization reaction time > graft polymerization reaction temperature.

#### 3.2.3. Variance Analysis and Discussion

The range analysis is simple but does not distinguish between data fluctuations caused by changes in experimental conditions and those due to experimental error. To estimate the extent of experimental error and to test whether the effects of experimental factors are significant, further analysis was performed using the analysis of variance and significance test (*P*-test), as shown in Table 3. From the analysis in the table, it can be seen that the F-value for the factor GMA concentration is significantly greater than 1 with a *p*-value of less than 0.05, indicating a significant effect of GMA concentration on the results. The F-value for the factor grafting time is relatively large, but with a *p*-value of more than 0.05, indicating a non-significant influence of grafting time on the results. The F-value for the factor grafting temperature is relatively small, with a *p*-value greater than 0.05, indicating a non-significant influence of grafting temperature on the results. The overall analysis indicates that the order of the factors is GMA concentration > grafting time > grafting temperature, which is consistent with the results of the range analysis mentioned above.

#### 3.2.4. The Amount of NMDG

**Figure 7:**
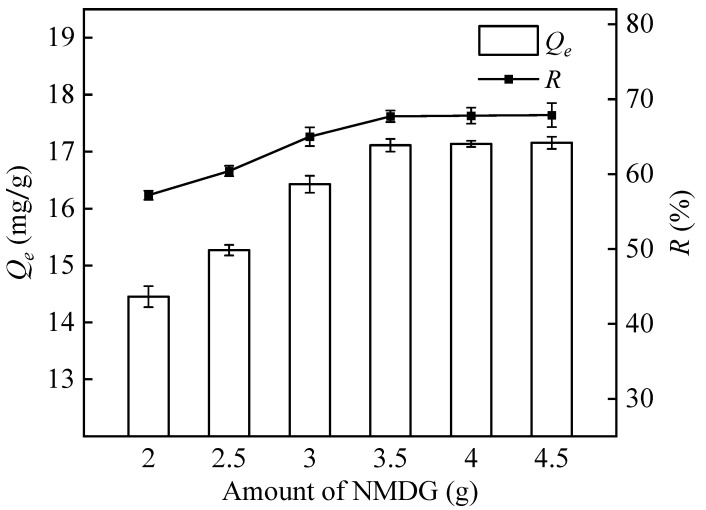
Effect of different amount of NMDG on adsorption capacity and removal rate.

NMDG includes many cy-o-dihydroxyl groups that have the ability to create stable cyclic esterification by chelating with H_3_BO_3_ or B(OH)_4_^−^. The amount of NMDG has a crucial role in influencing the ammoniation process [22]. Figure 7 demonstrates that as the amount of NMDG increases, the adsorption capacity of PP-g-GMA-NMDG for boron exhibits an early rise followed by a period of stability. Upon reaching an amount of 3.5 g, the adsorption capacity approached saturation, and subsequent increments in the quantity of NMDG did not yield a substantial enhancement in the adsorption efficacy. Hence, considering the cost of the functional monomer NMDG, the optimal dosage of NMDG in the ring opening amination reaction was determined to be 3.5 g.

### 3.3. Characterizations

#### 3.3.1. FT-IR

**Figure 8:**
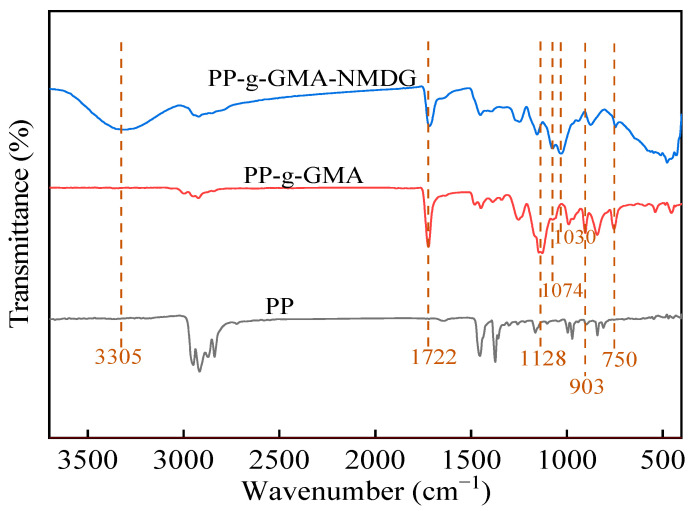
FT-IR spectra of PP, PP-g-GMA, PP-g-GMA-NMDG.

The FT-IR spectra of the prepared fibers are shown in Figure 8. The original PP fibers exhibit characteristic peaks at 2949 cm^−1^ and 2916 cm^−1^, corresponding to the asymmetric stretching vibrations of -CH_2_ and -CH_3_, respectively. The peaks at 1453 cm^−1^ and 1373 cm^−1^ represent symmetric stretching vibrations of -CH_2_ and symmetric deformation vibrations of -CH_3_, respectively, which confirms the composition of polypropylene [45]. Compared to PP fibers, PP-g-GMA fibers show stretching vibrational peaks of C=O and C-O-C groups at 1722 cm^−1^ and 1128 cm^−1^, respectively, indicating the presence of GMA. In addition, characteristic absorption peaks of epoxy groups are observed at 750 cm^−1^ and 903 cm^−1^. Peaks corresponding to the -OH, C-O, and C-N stretching vibrations of NMDG appear at 3305 cm^−1^, 1074 cm^−1^, and 1030 cm^−1^, respectively. Moreover, the strong characteristic peaks of epoxy groups at 750 cm^−1^ and 903 cm^−1^ in the PP-g-GMA-NMDG fibers diminish significantly, indicating the successful introduction of NMDG functional groups into the PP-g-GMA backbone. These results confirm the successful fabrication of PP-g-GMA-NMDG fibers.

#### 3.3.2. SEM

**Figure 9:**
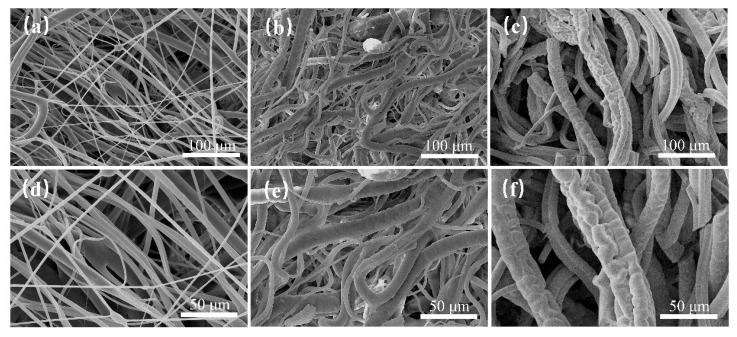
SEM diagram of (**a**,**d**) PP melt-blown fibers, (**b**,**e**) PP-g-GMA grafted with GMA concentration of 20%, and (**c**,**f**) PP-g-GMA-NMDG (20% GMA).

The SEM images in Figure 9 show the morphological structure of plasma-treated and untreated PP fibers at different magnifications. In the images, the surface of the untreated PP fibers appears relatively smooth and uniform. However, after plasma treatment and grafting with GMA, the surface of the PP-g-GMA fibers becomes rougher and has more functional groups with surface polarity. In addition, after the ring-opening amination reaction with NMDG, fractures appear on the surface of the PP-g-GMA-NMDG fibers accompanied by a significant increase in roughness and the formation of spiral patterns, which facilitate boron adsorption.

An elemental analysis was carried out to further confirm the graft polymerization of the modified PP fibers. The results are shown in Table 4. The elemental analysis shows a considerable amount of C on the surface of the PP fibers, which can be attributed to the C-H framework of PP. The introduction of GMA led to a significant increase in the O content due to the presence of epoxy groups on the surface of the PP-g-GMA fibers. After the amination ring-opening reaction, the O content of PP-g-GMA-NMDG was higher compared to PP-g-GMA, which is due to the hydroxyl groups in NMDG. These analyses indicate the successful preparation of PP-g-GMA-NMDG fibers, which is consistent with the results of the FT-IR analysis.

#### 3.3.3. Water Contact Angle

**Figure 10:**
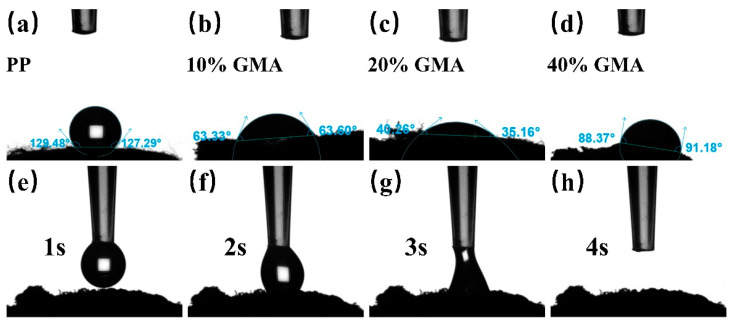
Contact angle of (**a**) PP melt-blown fibers; (**b**–**d**) PP-g-GMA grafted with GMA concentration from 10% to 40%; and (**e**–**h**) PP-g-GMA-NMDG (20% GMA).

The contact angle refers to the angle *θ* between the gas–liquid interface and the solid–liquid interface at the three-phase boundary of gas, liquid, and solid and serves as a measure of wettability. When *θ* < 90°, the solid surface is hydrophilic, which means that the liquid can easily wet the solid; *θ* > 90°, the solid is not wetted by the liquid and tends to move on the surface without entering capillaries [28]. Figure 10 shows the water contact angle of PP, PP-g-GMA, and PP-g-GMA-NMDG fibers. The water contact angle of PP fibers is 129° because they are non-polar and do not contain hydrophilic functional groups, resulting in high hydrophobicity. After grafting with different concentrations of GMA, the water contact angle of PP-g-GMA fibers decreases significantly, indicating increased hydrophilicity. With increasing GMA concentration, the water contact angle of PP-g-GMA fibers tends to decrease, followed by an increase, which is consistent with the trend of boron adsorption by PP-g-GMA-NMDG. GMA itself is hydrophobic and insoluble in water, but the plasma treatment can introduce hydroxyl groups into the PP, which then react with the GMA monomers and impart some hydrophilicity to the PP-g-GMA fibers. The water contact angle of PP-g-GMA-NMDG fibers is significantly lower, with deionized water spreading and wetting the surface immediately on contact. NMDG binds to the surface of the fibers after amination and significantly increases the hydrophilicity of the surface due to its high hydrophilicity.

### 3.4. Characterizations

#### 3.4.1. pH

**Figure 11:**
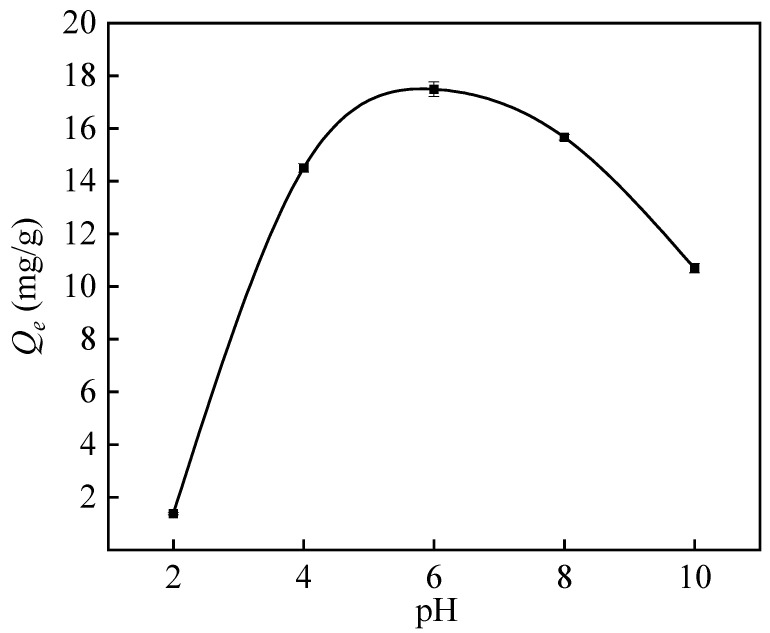
Effect of pH on the adsorption capacity of PP-g-GMA-NMDG.

In aqueous solutions, the predominant form of boron at lower pH is H_3_BO_3_, but at higher pH, the primary form of boron is B(OH)_4_^−^. The particular configurations of H_3_BO_3_ and B(OH)_4_^−^ are contingent upon the pH, temperature, and boron content of the solution. Boronic acid molecules undergo complexation processes and create numerous polynuclear compounds, such as B_3_O_3_(OH)_4_^−^, B_4_O_5_(OH)_4_^−^, and B_5_O_6_(OH)_4_^−^, in the pH range of 6 to 11 and at concentrations more than 0.025 mol/L [46]. Figure 11 shows that there is little variation in adsorption within the pH range of 4–8. In solutions with higher concentrations of H^+^, boric acid is mainly present in the form of H_3_BO_3_. Stable boric acid molecules exhibit good chelation with functional groups, and the negatively charged adsorbent enables good electrostatic interactions. However, H_3_BO_3_ has a weaker chelating ability compared to B(OH)_4_^−^ ions. Under acidic conditions, the degree of protonation of the adsorption sites is higher, which weakens the electrostatic interactions and inhibits chelation due to suppression by H^+^ ions [16]. With increasing pH, the concentration of B(OH)_4_^−^ ions increases, which leads to an increase in adsorption. Maximum adsorption, reaching 17.50 mg/g, occurs at pH 6, where chelation between the functional groups of the adsorbent and the borate ions is most stable. Beyond pH 6, when the solution passes from the neutral to the alkaline range, the various forms of boron present lead to poor chelation due to electrostatic repulsion. This shows that the pH value significantly influences the adsorption capacity of PP-g-GMA-NMDG. Therefore, a weakly acidic pH of 6 is preferred for optimal utilization as it offers stability, lower energy consumption, and environmental friendliness.

#### 3.4.2. Adsorption Time and Adsorption Kinetics

**Figure 12:**
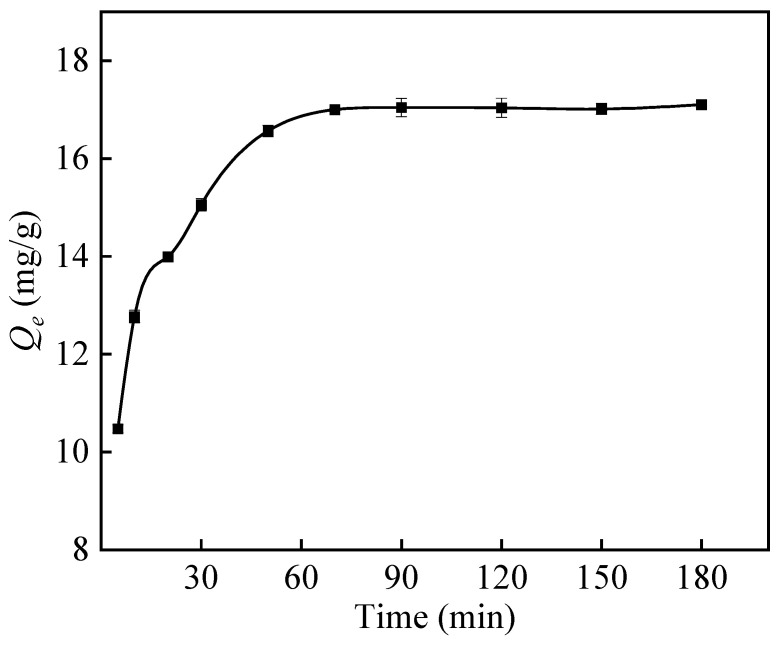
Effect of time on the adsorption capacity of PP-g-GMA-NMDG.

Figure 12 demonstrates that in the early stage of adsorption, when the ratio of fiber-to-surface area is high, boron diffuses quickly onto the surface of PP-g-GMA-NMDG and forms bonds with many available adsorption sites. This rapid diffusion phase has a brief duration, resulting in a quick adsorption rate. After 5 min, as adsorption advances and boron concentration declines, the number of accessible adsorption sites on the surface of PP-g-GMA-NMDG diminishes, leading to a reduction in adsorption rate and only minimal increases in adsorption capacity. Concurrently, the level of boron ions in the system decreases over time, resulting in a gradual stabilization of the adsorption capacity. The adsorption equilibrium is typically achieved within 70 min, and the maximal adsorption capacity is 17.04 mg/g.

**Figure 13:**
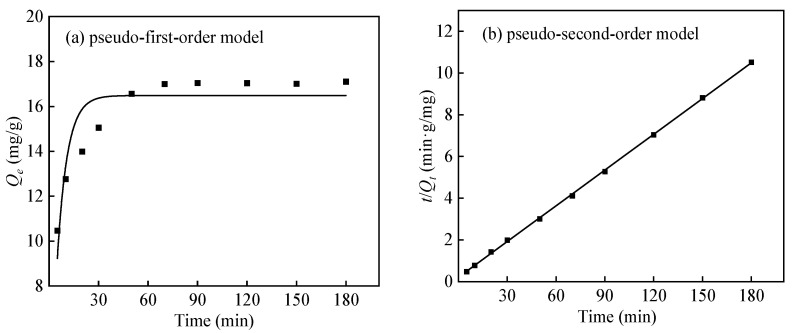
Kinetic fitting of boron adsorption by PP-g-GMA-NMDG.

Figure 13a,b display the pseudo-first-order and pseudo-second-order kinetic equations, respectively, for the adsorption of boron by PP-g-GMA-NMDG fibers. The fitting curves are also included. The outcomes of the fitting parameters are displayed in Table 5. The *R*^2^ value for the pseudo-first-order kinetic equation of PP-g-GMA-NMDG fibers is 0.799, while the *R*^2^ value for the pseudo-second-order kinetic equation is 0.999, as determined from the fitting results. A higher *R*^2^ value signifies a more accurate fit, and the equilibrium adsorption capacity derived from the pseudo-second-order kinetic equation is more closely aligned with the actual equilibrium adsorption capacity. Hence, the adsorption mechanism of boron by PP-g-GMA-NMDG fibers is better described by the pseudo-second-order kinetic equation, indicating that the rate of adsorption is directly proportional to the square of the concentration or pressure of the adsorbate. The pseudo-second-order kinetic model assumes that the rate of adsorption is governed by chemical adsorption mechanisms, which involve the sharing or transfer of electrons between the adsorbent and the adsorbate. This chemical adsorption involves the creation of chemical complexes between boron and the adsorbent, and the measured values are near the calculated values [16].

#### 3.4.3. Adsorption Isotherm and Adsorption Thermodynamics

**Figure 14:**
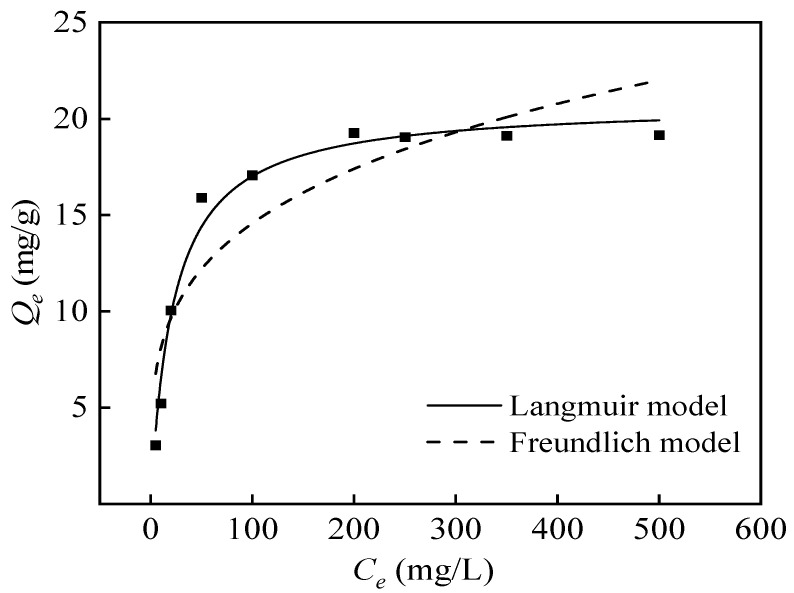
Langmuir and Freundlich fitting of adsorption isotherms.

**Figure 15:**
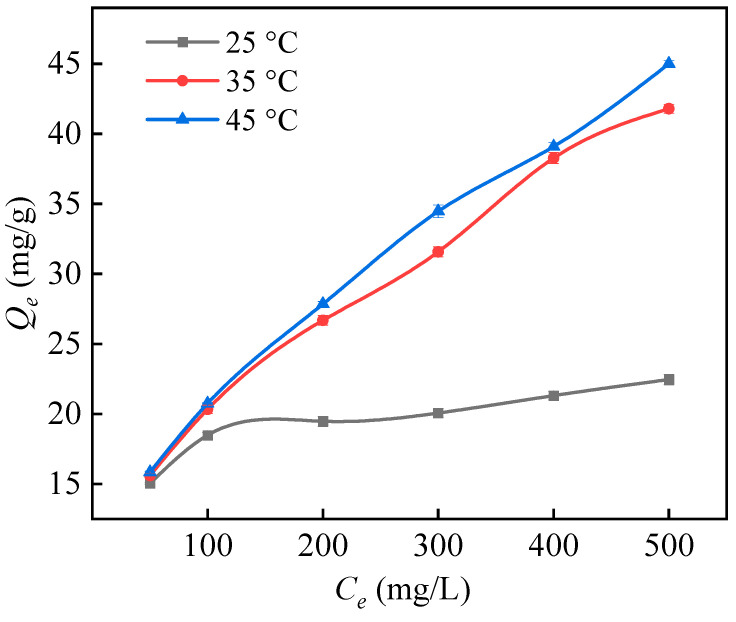
The influence of temperature on the adsorption capacity of PP-g-GMA-NMDG.

The connection between the initial concentration of the adsorption solution and the equilibrium adsorption capacity is shown in Figure 14. The Langmuir isotherm model explains the homogeneity of the adsorbent surface and a monolayer adsorption between the adsorbate and the adsorbent system, while the Freundlich isotherm curve describes surface heterogeneity and a multilayer adsorption of the adsorbate. Based on the data presented in Table 6, it is evident that the Langmuir model offers a more accurate fit, as indicated by a correlation coefficient (*R*^2^) of 0.981. This suggests that there is a monolayer chemical adsorption occurring between the adsorbent and boron, with adsorption sites evenly distributed on the surface. Thermodynamic tests on adsorption demonstrate that when the temperature increases, the adsorption capacity of PP-g-GMA-NMDG for boron likewise increases, as shown in Figure 15. Generally, raising the temperature can promote mass transfer and reduce solution viscosity, thereby increasing the diffusion rate of borate ions. Therefore, raising the solution temperature slightly is beneficial for the adsorption of boron [47].

#### 3.4.4. Adsorption Selectivity

**Figure 16:**
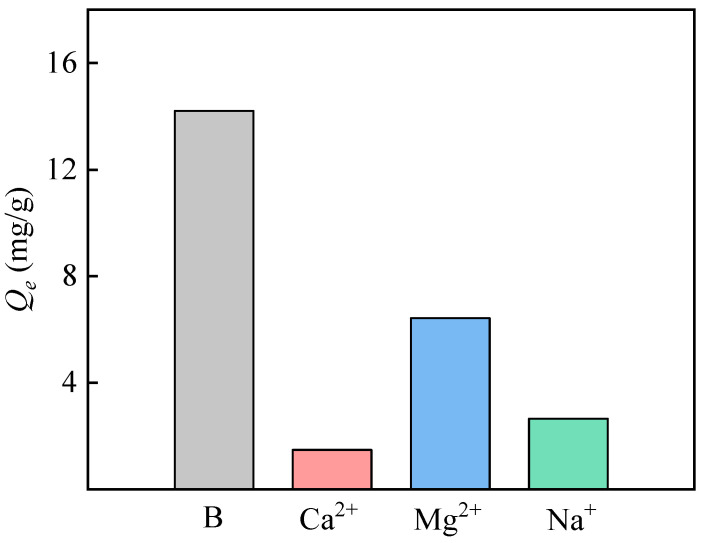
Adsorption capacity of PP-g-GMA-NMDG to boron and other coexistence ions in the solution containing multiple ions.

Salt lake brines are rich in ions, with high concentrations of Na^+^, Mg^2+^, and Ca^2+^ among them. The ability of PP-g-GMA-NMDG to adsorb boron fluctuates in the presence of interfering ions. Consequently, it is essential to research how other ions affect boron adsorption. The adsorption capacity of PP-g-GMA-NMDG was marginally lower in the multiple mixed solution than it was in the pure boron solution, as shown in Figure 16. In the meantime, PP-g-GMA-NMDG also adsorbed Na^+^, Mg^2+^, and Ca^2+^. Because Mg^2+^ complexes with -OH, it occupies some adsorption sites and establishes a competitive adsorption relationship, which explains why Mg^2+^ has a higher adsorption than Na^+^ and Ca^2+^. Since the extra salt ions balance the electrostatic and non-electrostatic forces, borate adsorption is only slightly affected, meaning that overall, the boron adsorption situation is still favorable. Furthermore, the solution’s metal ions may react to produce compounds such as magnesium hydroxide, which aids in boron adsorption [18]. The aforementioned findings demonstrate the superior selective adsorption capabilities of PP-g-GMA-NMDG fibers.

#### 3.4.5. Reusability

**Figure 17:**
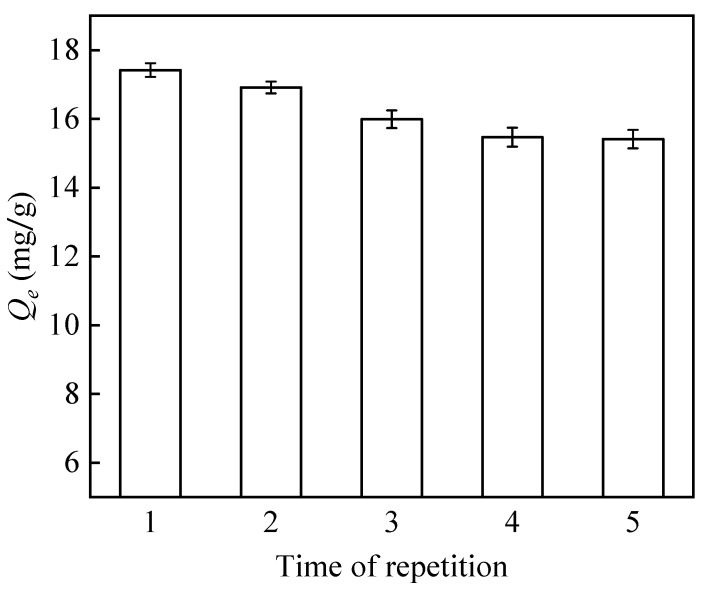
Reusability of PP-g-GMA-NMDG.

Practical applications greatly value reusability because it reduces expenses and conserves resources. The purpose of this experiment was to investigate the reusability of PP-g-GMA-NMDG fibers over five successive adsorption cycles. Figure 17 shows that the adsorption impact of PP-g-GMA-NMDG fibers on boron gradually decreases with an increase in the number of cycles. Even so, PP-g-GMA-NMDG maintained around 84.8% of its adsorption capabilities after being used five times in a row, suggesting that its ability to adsorb boron remained steady. The experimental results demonstrate that the PP-g-GMA-NMDG fibers can undergo many recycling processes and possess significant regenerative capabilities in practical applications.

#### 3.4.6. Comparison of Adsorption Capacity with Other Adsorbents

In this study, PP-g-GMA-NMDG had the highest adsorption capacity at 18.03 mg/g. According to current research on boron adsorbent materials, the adsorption capacity of the adsorbent utilized in this experiment is superior to that of other adsorbents. Table 7 provides support for this. This adsorbent possesses distinct benefits in terms of selectivity, reproducibility, and adsorption capacity. We selected PP melt-blown fibers as adsorbent substrates because of their inherent stability, low cost, and convenient recyclability. The produced adsorbent possesses numerous benefits and is the optimal selection for efficient boron extraction from salt lake brine.

## 4. Conclusions

In this work, plasma grafting polymerization was used to modify PP melt-blown fibers. The synthesis of the boron adsorbent PP-GMA-NMDG was illustrated through a range of characterization techniques. The process parameters for preparing the adsorbent were optimized using both single factor testing and orthogonal testing. A high-performance PP-GMA-NMDG material was synthesized, achieving an ideal boron adsorption capacity of 18.03 mg/g. The research on boron provided a deeper understanding of the adsorption process, including the mechanism, selectivity, and reusability of PP-GMA-NMDG. Overall, the PP-g-GMA-NMDG synthesized in this study demonstrates exceptional boron adsorption capabilities, favorable selectivity and reusability, and a greater adsorption capacity compared to alternative boron adsorbents. This indicates that it possesses significant potential for utilization in brine from boron-rich salt lakes and solutions for boron.

## Figures and Tables

**Table 1:** Experimental factors and levels.

Levels	Factors		
	A GMA Concentration (%)	B Graft Polymerization Reaction Time (min)	C Graft Polymerization Reaction Temperature (°C)
1	10	90	65
2	15	120	60
3	20	150	75

**Table 2:** Orthogonal experimental design table L_9_(3^4^) and experimental results.

Test Number	A	B	C	Blank	*Q_e_* (mg/g)
1	1	1	1	1	12.54
2	1	2	2	2	13.80
3	1	3	3	3	14.30
4	2	1	2	3	15.33
5	2	2	3	1	16.33
6	2	3	1	2	16.70
7	3	1	3	2	15.55
8	3	2	1	3	15.54
9	3	3	2	1	18.03
*k*1	13.54	14.47	14.93	15.63	
*k*2	16.12	15.22	15.72	15.35	
*k*3	16.38	16.34	15.40	15.06	
R*_k_*	2.83	1.87	0.79	0.58	
The sequence of the factors	ABC				
Optimal scheme	A3B3C2				

**Table 3:** Variance significance analysis.

Factors	Sum of Squares	DOF	MES	F Value	*p* Value	Significance
GMA concentration	15.51	2	7.76	29.53	0.03	*
graft polymerization time	5.61	2	2.81	10.69	0.09	
graft polymerization temperature	1.00	2	0.50	1.90	0.34	
Error	0.53	2	0.26			

* *p* < 0.05.

**Table 4:** Elemental analysis results.

Sample	Weight			Atomic		
	C (%)	N (%)	O (%)	C (%)	N (%)	O (%)
PP	94.22	0.00	5.78	95.60	0.00	4.40
PP-g-GMA	67.06	0.00	32.94	73.06	0.00	26.94
PP-g-GMA-NMDG	55.39	5.05	39.56	61.95	4.84	33.21

**Table 5:** Adsorption kinetics fitting parameters of PP-g-GMA-NMDG fibers.

*Q_e,exp_* (mg/g)	Pseudo-First-Order Model			Pseudo-Second-Order Model		
	*Q_e,cal_* (mg/g)	*k*_1_ (min^−1^)	*R* ^2^	*Q_e,cal_* (mg/g)	*k*_2_ (g·mg^−1^·min^−1^)	*R* ^2^
17.04	16.49	0.164	0.799	17.54	0.057	0.999

**Table 6:** Fitting coefficients of the Langmuir and Freundlich models.

Langmuir			Freundlich		
*K_L_*	*Q_m_* (mg/g)	*R* ^2^	*K* *_F_*	1/*n*	*R* ^2^
0.045	20.81	0.981	4.481	0.256	0.811

**Table 7:** Adsorption capacity of various adsorbents for boron.

Adsorbents	Substrate Material	Method	*Q_e_* (mg/g)	References
Red mud	Red mud	Batch equilibration technique	5.99	[48]
CACS	Activated carbons	Modification of multicomponent chelates	1.50	[49]
WTR	Waste tire rubber	Chemical modification	13.8	[50]
S-VBC-NMDG	Sulfur-based polymers prepared from sulfur and 4-vinylbenzyl chloride (VBC) revulcanization	Inverse-vulcanized	7.2	[51]
PP-g-GMA-NMDG	PP melt-blow fibers	Plasma grafting polymerization	18.03	This work

## Data Availability

Data are contained within the article.
